# Total Variation Regularization of Matrix-Valued Images

**DOI:** 10.1155/2007/27432

**Published:** 2007-06-07

**Authors:** Oddvar Christiansen, Tin-Man Lee, Johan Lie, Usha Sinha, Tony F. Chan

**Affiliations:** ^1^Department of Mathematics, Faculty of Mathematics and Natural Sciences, University of Bergen, Bergen 5008, Norway; ^2^Medical Imaging Informatics Group, Department of Radiological Sciences, University of California, Los Angeles, 90024 CA, USA; ^3^Division of Physical Sciences, College of letters science, University of California, Los Angeles, 90095 CA, USA

## Abstract

We generalize the total variation restoration model, introduced by Rudin, Osher, and Fatemi in 1992, to matrix-valued data, in particular, to diffusion tensor images (DTIs). Our model is a natural extension of the color total variation model proposed by Blomgren and Chan in 1998. We treat the diffusion matrix *D* implicitly as the product *D* = *LL* ^*T*^, and work with the elements of *L* as variables, instead of working directly on the elements of *D*. This ensures positive definiteness of the tensor during the regularization flow, which is essential when regularizing DTI. We perform numerical experiments on both synthetical data and 3D human brain DTI, and measure the quantitative behavior of the proposed model.

## 1. INTRODUCTION

Image processing methods using variational calculus and partial differential equations (PDEs) have been popular for a long time in the image processing research community. Among popular PDE methods are the anisotropic diffusion method proposed by Perona and Malik [1], the total variation method introduced by Rudin et al. [2], and various methods related to these [3–7]. Many of these methods were originally introduced for scalar-valued (gray-scale) images, and were later generalized to vector-valued (color) images.

**Figure 1:**
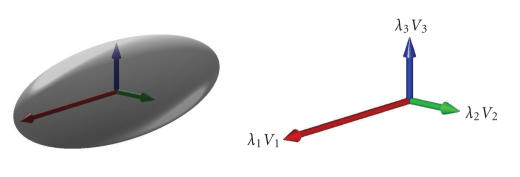
The diffusion matrix *D* can be represented by a diffusion ellipsoid, where the semiaxes are spanned by the eigenvectors {*V*_*i*_}^3^_*i* = 1_ of *D*, and the length of each semiaxis is given by the eigenvalues {*λ*_*i*_}^3^_*i* = 1_. In this illustration, the diffusion is anisotropic. The principal diffusion direction is along eigenvector *V*_1_.

During the last decade or so, a new magnetic resonance modality called diffusion tensor imaging (DTI) has been extensively studied [8–13]. Using DTI, it is possible to study anatomical structures like the nerve fibers in the human brain noninvasively. The DTI images are matrix valued. In each voxel of the imaging domain, we construct a *diffusion tensor* (i.e., *diffusion matrix*) *D* based on a series of *K* direction-specific MR measurements {*S*_*k*_}^*K*^_*k* = 1_. The matrix *D* ∈ *R* ^3 × 3^ is a symmetric, positive definite matrix

(1) $$D=V\text{Λ}V^{-1},$$

where *V* is an orthogonal matrix, and Λ is a diagonal matrix with positive elements. We may look at the diffusion matrix as a hyperellipse where the eigenvectors {*V*_*i*_}^3^_*i* = 1_ span the ellipsoid, and the corresponding eigenvalues {*λ*_*i*_}^3^_*i* = 1_ determine the length of each semiaxis (see Figure 1). It is customary to arrange the eigenvalues in decreasing order. By the diffusion tensor model we assume that the set of images {*S*_*k*_}^*K*^_*k* = 1_ is related to the nonweighted image *S*_0_ by the Stejskal-Tanner equation [14, 15]

(2) $$S_{k}=S_{0}e^{-bg_{k}^{T}Dg_{k}},\;\;\;\;k=1,2,\ldots ,K.$$

Here *g*_*k*_ ∈ *R* ^3^ denotes the direction associated with *S*_*k*_, and *b* > 0 is a scalar which among other factors depends on the acquisition time and the strength of the magnetic field [16]. Since *D* ∈ *R* ^3 × 3^ is symmetric, it has six degrees of freedom. Thus at least six measurements {*S*_*k*_}^6^_*k* = 1_ are required to estimate the tensor, as well as the nonweighted measurement *S*_0_. The tensor *D* can be estimated from (2). In the case of more than six measurements *S*_*k*_, we can estimate *D* by, for example, a least-squares minimization. From the eigenvalue decomposition of the diffusion tensor, we can reveal properties like the dominant diffusion direction and the anisotropy of diffusing water molecules [17]. This information can be used to construct maps of the anatomy of the brain.

From the developments in DTI, a need for robust regularization methods for matrix-valued images has emerged. As far as the authors are aware of, there exists no state-of-the-art method for regularization of tensor-valued images, although many methods have been proposed [18–21].

All measurements {*S*_*k*_}^*K*^_*k* = 1_ contain noise, which degrades the accuracy of the estimated tensor. Compared with conventional MR, direction-sensitive acquisitions have a lower signal-to-noise ratio (SNR). Thus the gradient weighted images {*S*_*k*_}^*K*^_*k* = 1_ contain more noise than *S*_0_. There are several ways to increase the accuracy of the estimated tensor. The most intuitive way is to make an average of a series of repeated measurements. Alternatively, we can increase the number of gradient directions. An obvious disadvantage of both of these approaches is the increased scanner time. Perhaps a better way to improve the quality of the tensor is by postprocessing the data. In this paper, we follow this approach, by introducing a regularization method for tensor-valued data.

Since *D* models diffusion, regularization methods in DTI must preserve the positive definiteness of *D*. A positive definite matrix has only positive eigenvalues, which is necessary from the physical modeling perspective. In a minimization method for regularization of the tensor data, one possible way to ensure positive definiteness would be to impose a constraint on the minimization problem. Then the constrained problem would have a solution which is on the manifold of positive definite matrices. Regularization of tensor-valued data constrained to manifolds has been studied during the last couple of years, see [22–24]. We however follow a different strategy. Using essentially the same idea as Wang et al. did in a slightly different setting, we treat *D* implicitly by writing *D* as the product *D* = *LL* ^*T*^, where *L* is a lower triangular matrix [18]. Every symmetric positive definite (SPD) matrix has a factorization on this form. We will in this work develop a regularization method for diffusion tensor images by generalizing methods previously developed for scalar- and vector-valued images [2, 25].

Before we go into details of the proposed method, we briefly introduce the total variation (TV) methods for scalar- and vector-valued images. During the last 15 years or so, TV models have undergone extensive studies, initiated by the work of Rudin, Osher, and Fatemi (ROF) [2].

Define the total variation (TV) seminorm for scalar-valued data as

(3) $$\text{TV}\left[u\right]=\int_{\text{Ω}}\left|\nabla u\right|dx.$$

Throughout this paper, ∇ denotes the spatial gradient, while ∇ ⋅ denotes the divergence operator. In the ROF model, the TV seminorm with an added *L*_2_ fidelity norm is minimized

(4) $$\underset{u}{\min}\left\{G\left(u,f,\lambda \right)=\text{TV}\left[u\right]+\frac{\lambda }{2}{\left\|u-f\right\|}_{2}^{2}\right\}.$$

Note that we can write the functional *G* more abstractly as

(5) $$G\left(u,f,\lambda \right)=R\left(u\right)+\frac{\lambda }{2}F\left(u,f\right),$$

where *R*(*u*) is a regularization functional and *F*(*u, f*) is a fidelity functional. The regularization term is a geometric functional measuring smoothness of the estimated solution. The fidelity term is a measure of fitness of the estimated solution compared to the input data. It is customary to measure the fidelity in the sense of least squares. Equation (4) has the corresponding Euler-Lagrange equation

(6) $$\partial_{u}G=-\nabla \cdot \left(\frac{\nabla u}{\left|\nabla u\right|}\right)+\lambda \left(u-f\right).$$

We can find a minimum of (4) by searching for a steady state of

(7) $$\frac{\partial u}{\partial t}=-\partial_{u}G,$$

which is the way the ROF model was first formulated [2]. Alternatively, we can directly attack the zero of the Euler-Lagrange equation

(8) $$-\partial_{u}G=0,$$

for example, by a fixed-point iteration [26]. This is in general less time consuming than solving the equation using the method of steepest descent, but more tedious to carry out numerically. When we generalize the method to matrix-valued images, we solve the minimization problem by the method of steepest descent. Various methods have been proposed to generalize the ROF model to vector-valued image regularization. Among the successful methods, we find the color TV model developed by Blomgren and Chan [25] and the model by Sapiro [27]. Blomgren and Chan [25] generalized the ROF model to color (vector) image regularization using a set of coupled equations

(9) $$\left\{\frac{\partial u_{i}}{\partial t}=\alpha_{i}\nabla \cdot \left(\frac{\nabla u_{i}}{\left|\nabla u_{i}\right|}\right)-\lambda \left(u_{i}-f_{i}\right),\;i=1,2,3\right\}$$

with

(10) $$\begin{array}{c} \alpha_{i}=\frac{\text{TV}\left[u_{i}\right]}{\text{TV}\left[\text{u}\right]},\;\;\;\;i=1,2,3,\\ \text{TV}\left[\text{u}\right]=\sqrt{\sum_{i=1}^{3}\text{TV}{\left[u_{i}\right]}^{2}}. \end{array}$$

The weight *α*_*i*_ in (9) acts as a coupling between the geometric part of the three image channels. In this work, we extend in a natural way the color TV model of Blomgren and Chan to a matrix TV model. However, the method we propose is not restricted to our choice of regularization functional (TV). For a detailed treatment of TV regularization methods we refer the reader to the recent book by Chan and Shen [5].

In Section 2, we define the minimization problem that we propose to solve, and arrive at the Euler-Lagrange equations corresponding to this minimization problem. We perform numerical experiments in Section 3, before we finish the paper in Section 4 with a conclusion. Details on the Euler-Lagrange equation and the numerical implementation are given in the appendix at the end of the paper.

## 2. MINIMIZATION PROBLEM

In this section, we introduce the functional that we minimize in order to regularize tensor-valued data. Let *L* be a lower triangular matrix. We define *D* by

(11) $$D=LL^{T}.$$

This has immediate implications on *D*: symmetry, positive definiteness, and orthogonality of eigenvectors. These properties are required by the diffusion tensor model. Thus (11) is a natural choice. We define *ℓ*_*ij*_ as the element in the *i*th row and *j*th column of *L*. The elements *d*_*ij*_ are defined in the same manner.

Let us look at the algebraic equation expressing *D* as a function of *ℓ*_*ij*_. We derive the expressions for *D* ∈ *R* ^3 × 3^ ⊂ SPD. We explicitly write out the matrix multiplication (11),

(12) $$D=\left(\begin{array}{ccc} \ell_{11}^{2} & \ell_{11}\ell_{21} & \ell_{11}\ell_{31}\\ \ell_{11}\ell_{21} & \ell_{21}^{2}+\ell_{22}^{2} & \ell_{21}\ell_{31}+\ell_{22}\ell_{32}\\ \ell_{11}\ell_{31} & \ell_{21}\ell_{31}+\ell_{22}\ell_{32} & \ell_{31}^{2}+\ell_{32}^{2}+\ell_{33}^{2} \end{array}\right).$$

In our proposed model, we solve a minimization problem in terms of *ℓ*_*ij*_. For each unique *ℓ*_*kl*_, we minimize

(13) $$\underset{\ell_{kl}}{\min}\left\{\sqrt{\sum_{ij}\text{TV}{\left[d_{ij}\left(\ell_{kl}\right)\right]}^{2}}+\frac{\lambda }{2}\sum_{ij}{\left\|d_{ij}-{\hat{d}}_{ij}\right\|}_{2}^{2}\right\},$$

where {*kl*} ∈ {11, 21, 22, 31, 32, 33} and ${\hat{d}}_{ij}$ denotes the elements of the tensor estimated from the noisy data. As in the scalar model, the functional (13) has the abstract form (5). The scalar ROF (TV − *L*_2_) functional is convex. But when we introduce the factorization (11) into the model, we cannot expect the functional (13) to be convex or even quasiconvex. However, from numerical experiments where we used different (random) intial conditions we ended up with almost exactly the same solution. This means that even though we are not able to prove that the functional is convex, we have indications that it is at least quasiconvex.

We note that minimizing the functional (13) is related to the functional used by Wang et al. [18]. Apart from the fact that they simultaneously estimate and regularize the tensor, there are fundamental differences between our proposed regularization functional and the functional proposed by Wang et al. Even though we represent the diffusion matrix on the form of a Cholesky factorization, we regularize the elements of the full diffusion tensor *D*, while Wang et al. regularize the elements of the lower triangular matrix *L*. Intuitively, regularizing the elements of *D* is more direct than regularizing the elements of *L*. We highlight the difference between Wang's method and our proposed method by a numerical simulation in a simplified setting in Section 3. In addition, the method proposed in this paper has a coupling between all elements of *D* in the regularization PDE, while the method proposed by Wang et al. does not have such a coupling between the channels.

We also note that the functional (13) is chosen mainly because of the good properties of the corresponding scalar- and vector-valued functionals [2, 25], with edge preservation as the most prominent property. Depending on the application at hand, other functionals might be considered as alternatives. The framework developed in this paper is however applicable for other regularization functionals as well.

### 2.1. Euler-Lagrange equations

In this section, we derive the Euler-Lagrange equations corresponding to the minimization functional (13). We first differentiate the fidelity functional

(14) $$\begin{array}{c} \frac{\partial F}{\partial \ell_{kl}}=\frac{\partial }{\partial \ell_{kl}}\sum_{ij}{\left\|d_{ij}-{\hat{d}}_{ij}\right\|}_{2}^{2}\\ =2\sum_{ij}\left(d_{ij}-{\hat{d}}_{ij}\right)\frac{\partial d_{ij}}{\partial \ell_{kl}}. \end{array}$$

We differentiate *D* with respect to *ℓ*_*kl*_, for example

(15) $$\frac{\partial D}{\partial \ell_{11}}=\left(\begin{array}{ccc} 2\ell_{11} & \ell_{21} & \ell_{31}\\ \ell_{21} & 0 & 0\\ \ell_{31} & 0 & 0 \end{array}\right).$$

The other derivatives follow the same pattern. Writing out (14), we find the derivative of the fidelity functional,

(16) $$\begin{array}{l} \frac{\partial F}{\partial \ell_{kl}}=2[\left(d_{11}-{\hat{d}}_{11}\right)\frac{\partial d_{11}}{\partial \ell_{kl}}+2\left(d_{21}-{\hat{d}}_{21}\right)\frac{\partial d_{21}}{\partial \ell_{kl}}\\ \;\;\;\;\;\;\;\;\;\;\;\;\;\;\;\;\;\;\;\;\;\;+\left(d_{22}-{\hat{d}}_{22}\right)\frac{\partial d_{22}}{\partial \ell_{kl}}+2\left(d_{31}-{\hat{d}}_{31}\right)\frac{\partial d_{31}}{\partial \ell_{kl}}\\ \;\;\;\;\;\;\;\;\;\;\;\;\;\;\;\;\;\;\;\;\;\;+2\left(d_{32}-{\hat{d}}_{32}\right)\frac{\partial d_{32}}{\partial \ell_{kl}}+\left(d_{33}-{\hat{d}}_{33}\right)\frac{\partial d_{33}}{\partial \ell_{kl}}], \end{array}$$

where {*d_ij_*}^3^_*i* = 1, *j* = 1_ denote the elements of the matrix *D*. We differentiate the regularization functional in (13). Define the total variation norm of a matrix *D* ∈ *R* ^3^ × *R* ^3^ as

(17) $$\begin{array}{l} \text{TV}\left[D\right]=(\text{TV}{\left[d_{11}\left(\ell_{ij}\right)\right]}^{2}+2\text{TV}{\left[d_{21}\left(\ell_{ij}\right)\right]}^{2}\\ \;\;\;\;\;\;\;\;\;\;\;\;\;\;\;\;\;\;\;\;\;\;\;+\text{TV}{\left[d_{22}\left(\ell_{ij}\right)\right]}^{2}+2\text{TV}{\left[d_{31}\left(\ell_{ij}\right)\right]}^{2}\\ \;\;\;\;\;\;\;\;\;\;\;\;\;\;\;\;\;\;\;\;\;\;\;{+2\text{TV}{\left[d_{32}\left(\ell_{ij}\right)\right]}^{2}+\text{TV}{\left[d_{33}\left(\ell_{ij}\right)\right]}^{2})}^{1/2}. \end{array}$$

This is a straightforward generalization of the total variation norm of a vector [25].

Using the chain rule, we find the derivatives of the regularization functional to be

(18) $$\frac{\partial R}{\partial \ell_{kl}}=-\sum_{ij}\alpha_{ij}\nabla \cdot \left(\frac{\nabla d_{ij}}{\left|\nabla d_{ij}\right|}\right)\frac{\partial d_{ij}}{\partial \ell_{kl}},$$

with

(19) $$\alpha_{ij}=\frac{\text{TV}\left[d_{ij}\right]}{\text{TV}\left[D\right]}.$$

Note here that this derivative is essentially similar to the derivative in the color TV model of Blomgren and Chan [25], but with the important difference that we represent the diffusion matrix by its Cholesky factors.

In the next section, we perform numerical simulations using the method proposed in this paper. We give more details on the Euler-Lagrange equations in the appendix, which also contains some details on the numerical implementation of the model.

## 3. NUMERICAL EXPERIMENTS

In this section we perform numerical experiments on synthetically constructed tensor fields and real tensor fields from a human brain. The numerical implementation of the method is briefly discussed in the appendix.

For the synthetical fields we have constructed clean tensor fields, which are degraded with noise with a prior known distribution. Thus, we are able to measure how well the method performs on synthetical data. For the real human brain DTI, the “true” solution is of course not known in advance. In this case, we measure the performance of the method in terms of a reference solution where a large series of acquisitions are averaged. This is explained in detail in Section 3.3. For the numerical implementation of the method and some of the visualizations, we have used Matlab [28].

### 3.1. Synthetical tensor fields

**Figure 2:**
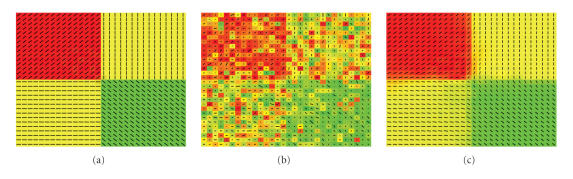
A synthetically produced purely anisotropic tensor field with four distinct regions is degraded with normally distributed noise. The noisy field is then processed with our proposed method: (a) the clean vector field *D*_0_; (b) the noisy field $\hat{D}$; (c) the recovered field *D*.

In the first numerical experiment, displayed in Figure 2, we test the performance of the proposed method on a simple tensor field. This field is mapping a square domain Ω ⊂ *R* ^2^, with four distinct regions, to *R* ^2 × 2^. We construct the clean tensor-valued data by prescribing the eigenvalues and corresponding eigenvectors. The values of each element of *L* is in the range [0,1]. Then we add normally distributed noise *η*(*σ*) to each element of the Cholesky factorization of the matrix, that is, $\hat{L}=L+\eta \left(\sigma \right)$. Finally, the degraded diffusion tensor is constructed by $\hat{D}=\hat{L}{\hat{L}}^{T}$. The noise levels in the simulations in Figures 2 and 4 are given by *σ* = 0.35 and *σ* = 0.25, respectively. The time step is Δ*t* = 0.001. Note that the discontinuities in the data are preserved in the solution, that is, the edge preserving property of scalar and vector-valued TV flow is kept in the proposed matrix-valued flow. In the first example, the diffusion is anisotropic in the whole domain. To show how the proposed method differentiates between isotropic and anisotropic regions we show a similar example where one of the four regions is interchanged with an isotropic region. The isotropic region is constructed by considering the orthogonal matrix *Q* from the *QR* factorization of a random 2 × 2 matrix. The columns of the matrix *Q* are considered to be the eigenvectors of the diffusion tensor. We specify two random numbers in the range [0,1] as the eigenvalues of the tensor. Thus the diffusion is random in the isotropic region. As we can observe from these two numerical examples on synthetical data, edges are preserved in the regularized images. We observe that even when the noise level is high, we are able to reconstruct an image which is close to the true noise-free image.

From these numerical experiments on synthetical data we see that the proposed method gives encouraging results. Similarly as in the scalar- and vector-valued settings, edges are well preserved. We further investigate the edge preservation in the next experiment.

### 3.2. Qualitative experiments

**Figure 3:**
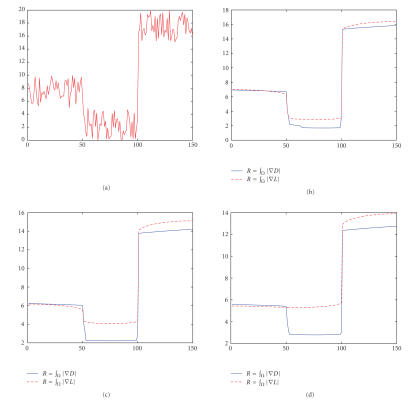
A simple 1D example showing the qualitative behavior of the model for regularizers *∫*_Ω_ |∇ *L*| and *∫*_Ω_ |∇ *D*|. The noisy signal in (a) is processed with both flows. Subfigures (b), (c), and (d) are snapshots during the flow at the three times *t* = 8, *t* = 16, and *t* = 24.

**Figure 4:**
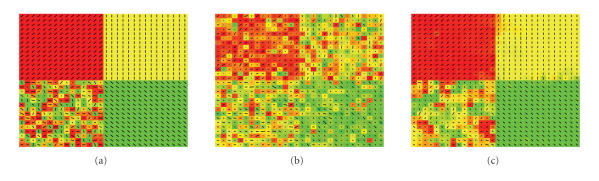
Visualization of (a) the true vector field, (b) the noisy field, and (c) the recovered field. In this example, the tensor field is isotropic in the lower-left corner, anisotropic in the other parts.

To highlight the qualitative differences between regularizing the elements of the tensor *D* and the elements of the Cholesky factors *L*, we have constructed a simple numerical example in 1D. We have removed the fidelity measure from the model, thus the method is in this setting merely a diffusion filter. Thus we have simplified the model in such a way that we can study the qualitative behavior of the two regularization filters in the same setting (see Figure 3). From this example, we clearly see that when we regularize *D* the edges are better preserved than when we regularize *L*. Note that Wang et al. regularize the Cholesky factors [18].

**Figure 5:**
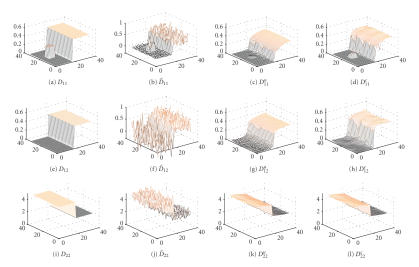
A noisy 2D tensor field is regularized. In this example, the smallest parts of the signal are not easily discriminated from the noise.

We also present a numerical example in 2D where we solve the PDEs first as an uncoupled system, that is, by employing the weighting factors *α*_*ij*_ = 1, and then as a coupled system where we use the weighting factors from (10). We denote the clean field by *D*, the noisy field by $\hat{D}$, the field regularized with the uncoupled system by *D* ^*u*^ and the field regularized with the coupled system by *D* ^*c*^. In Figures 5(a)–5(d), we show the elements *D*_11_, ${\hat{D}}_{11}$, *D* ^*u*^_11_, and *D* ^*c*^_11_, respectively. Subindexes denote the elements of the matrix field. Figures 5(e)–5(h) show the elements *D*_12_, ${\hat{D}}_{12}$, *D* ^*u*^_12_, and *D* ^*c*^_12_, while Figures 5(i)–5(l) show the element *D*_22_, ${\hat{D}}_{22}$, *D* ^*u*^_22_, and *D* ^*c*^_22_. From Figure 5, we observe that the uncoupled system does not discriminate the noise from the weak signal. The coupled system on the other hand better discriminates the noise from the weak signal. A similar 1D example is shown by Blomgren and Chan using the color TV model [25].

In the next section, we go one step further and process real human brain DTMRI.

### 3.3. Human brain DTMRI

We also perform numerical experiments on DTMRI acquisitions of a healthy human brain from a volunteer. The human subject data is acquired using a 3.0 T scanner (Magnetom Trio, Siemens Medical Solutions, Erlangen, Germany) with an 8-element head coil array and a gradient subsystem with the maximum gradient strength of 40 mT/m and maximum slew rate of 200 mT/m/ms. The DTI data is based on spin-echo single-shot EPI acquired utilizing generalized autocalibrating partially parallel acquisitions (GRAPPA) technique with acceleration factor of 2 and 64 reference lines. The DTI acquisition consists of one baseline EPI and six diffusion weighted images (b-factor of 1000 s/mm^2^) along the following gradient directions: $\begin{array}{l} G_{1}=1/\sqrt{2}{\left[1,0,1\right]}^{T},\;G_{2}=1/\sqrt{2}{\left[-1,0,1\right]}^{T},\;G_{3}=1/\sqrt{2}{\left[0,1,1\right]}^{T},\;G_{4}=1/\sqrt{2}{\left[0,1,-1\right]}^{T},\\ G_{5}=1/\sqrt{2}{\left[1,1,0\right]}^{T},\;G_{6}=1/\sqrt{2}{\left[1,1,0\right]}^{T} \end{array}$. Each acquisition has the following parameters: TE/TR/averages is 91 ms/10000 ms/2, FOV is 256 mm × 256 mm, slice thickness/gap is 2 mm/0 mm, acquisition matrix is 192 × 192 pixels, and partial Fourier encoding is 75%.

For validation of the proposed regularization method on real data, we construct a reference solution D* by averaging 18 replications. Each replication consists of six-direction weighted and one nonweighted acquisitions. This reference solution is compared to solutions where averages of two, four, and six replications are postprocessed with the proposed regularization method. As a measure of the distance between the reference solution and the processed solution, we use the following metric:

(20) $$\begin{array}{l} m\left(D,D^{*}\right)=({\left[d_{11}-d_{11}^{*}\right]}^{2}+2{\left[d_{12}-d_{12}^{*}\right]}^{2}\\ \;\;\;\;\;\;\;\;\;\;\;\;\;\;\;\;\;\;\;\;\;\;\;\;\;\;\;\;\;\;+2{\left[d_{13}-d_{13}^{*}\right]}^{2}+{\left[d_{22}-d_{22}^{*}\right]}^{2}\\ \;\;\;\;\;\;\;\;\;\;\;\;\;\;\;\;\;\;\;\;\;\;\;\;\;\;\;\;\;\;{+2{\left[d_{23}-d_{23}^{*}\right]}^{2}+{\left[d_{33}-d_{33}^{*}\right]}^{2})}^{1/2}. \end{array}$$

**Figure 6:**
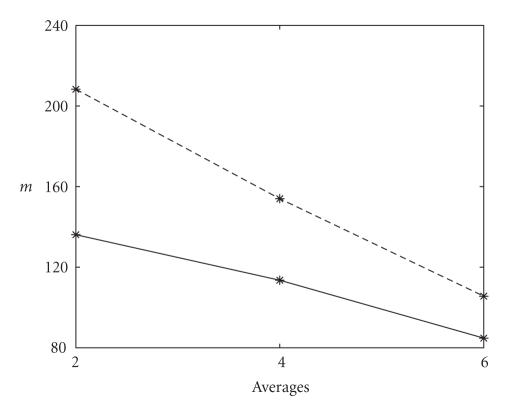
Comparison of *m*(*D*, D*) for the original tensors (dashed) and the regularized tensors (solid) versus the number of averaged acquisitions.

**Figure 7:**
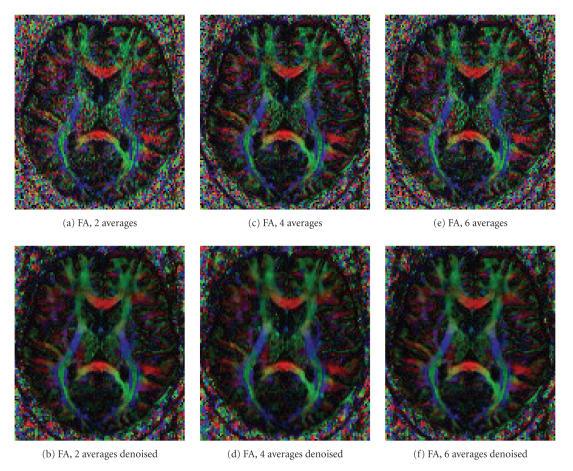
Color-coded fractional anisotropy (FA) maps constructed from averages of two (a), four (c), and six (e) acquisitions, and the corresponding denoised maps (b), (d), and (f).

**Figure 8:**
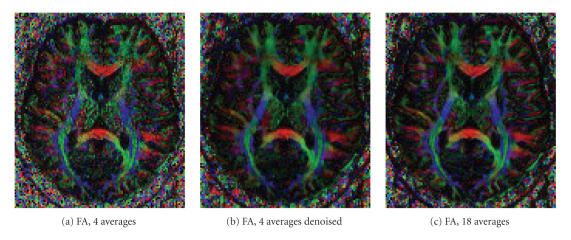
The noisy 4-average acquisition (a) is compared with the denoised acquisition (b) and a reference solution at 18 averages.

For each simulation, we report the regularization parameter *λ* and the metric *m*(⋅, ⋅) in Table 1 and in Figure 6. We display the result before and after applying the proposed method on real DTMRI data in Figures 7 and 8. In the figures, we display a 2D slice of an RGB direction encoded fractional-anisotropy (FA) measure defined by

(21) $$\text{FA}=\sqrt{\frac{3}{2}\frac{{\left(\bar{\lambda }-\lambda_{1}\right)}^{2}+{\left(\bar{\lambda }-\lambda_{2}\right)}^{2}+{\left(\bar{\lambda }-\lambda_{3}\right)}^{2}}{\lambda_{1}^{2}+\lambda_{2}^{2}+\lambda_{3}^{2}}},$$

where $\bar{\lambda }=\left(\lambda_{1}+\lambda_{2}+\lambda_{3}\right)/3$. The FA measure is direction-encoded as described by Pajevic and Pierpaoli [29]. We use the DTMRI software DTIStudio to construct the visualizations [30]. In the figures, we show the color-coded FA.

The noise level is different for each simulation due to the varying number of acquisitions. Consequently, the regularization parameter *λ* is different for each simulation. However, for clinical applications, the regularization parameter is estimated once for each imaging protocol. When this is done, the same regularization parameter can be used for subsequent applications of the same imaging protocol.

### 3.4. Human brain ROI study

Since our algorithm regularizes the tensor field, we focus on the evaluation of the tensor field, and the derived scalar FA map. However, we note that from the processed tensor field, we may reconstruct the corresponding diffusion weighted images {*S*_*i*_}^6^_*i* = 1_ by (2). There are obvious visual improvements in the processed diffusion weighted images compared to the noisy diffusion weighted images. Edges are preserved and noise is suppressed. Quantitatively, the mean and standard deviation at certain homogeneous regions of interests (ROIs) show significant improvements. We will now assess the visual and quantitative improvements in terms of the denoised tensors.

**Figure 9:**
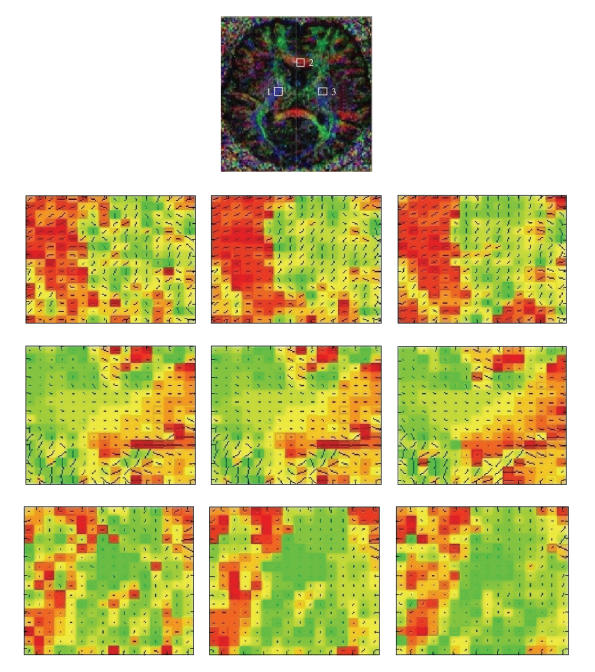
ROI study. Top image shows the ROIs that we use. The second row from left to right: the noisy (4-average) data, denoised data with *λ* = 13, and clean (18-average) data of ROI 1. The third row from left to right: the noisy (4-average) data, denoised data with *λ* = 13, and clean (18-average) data of ROI 2. The fourth row from left to right: the noisy (4-average) data, denoised data with *λ* = 13, and clean (18-average) data of ROI 3.

For qualitative evaluation, we select three regions of interest (ROIs) from one slice of the acquired images, with a 15-by-15 voxel size. We plot the 2D projection of the eigenvector corresponding to the major eigenvalue in Figure 9. From Figure 9, we can clearly see that our method preserves discontinuities (edges) in the tensor field, while it smooths the tensor field in homogeneous regions. The denoised tensor field from the 4-average acquisition is close to the tensor field obtained from the 18-average acquisition.

For quantitative measures, we use the average deviation angle (ADA) index of Chen and Hsu to measure if the tensor field undergoes gradual changes or sharp turns [21]. The PDE filtering is performed after the tensors are computed, so we use the angle deviation in adjacent voxels as a measure of its performance instead of normalized magnitude of diffusion tensor error (NMTE) index [21]. Denote the eigenvector corresponding to the largest eigenvalue by V*. Define the ADA by

(22) $$\text{ADA}=\frac{\text{Δ}\alpha_{i-1}+\text{Δ}\alpha_{i+1}+\text{Δ}\alpha_{j-1}+\text{Δ}\alpha_{j+1}+\text{Δ}\alpha_{k-1}+\text{Δ}\alpha_{k+1}}{6},$$

where, for example, $\text{Δ}\alpha_{i-1}=\cos^{-1}\left(\left|\left(V_{ijk}^{*},V_{i-1jk}^{*}\right)\right|\right)$. We note that we use the absolute value of the inner product (⋅, ⋅) to accommodate antisense directional vectors. A small change in direction from one voxel to its neighbor gives a small ADA, while a large change in direction gives a large ADA.

After masking the background, we compute the average ADA within the brain, and call it the global ADA. From Table 2, we see that the global ADA of the data is reduced from 12.31 to 6.27 by the denoising algorithm, whereas the 18-average clean data has an ADA of 6.65. With a higher data fidelity requirement (when *λ* is larger, e.g., 20), the smoothing is not very aggressive and the ADA is not as close as when *λ* = 13. When *λ* is less than 13 (data not shown here), the smoothing is excessive and the ADA values fall well below the ADA of the 18-average data. From this information, we conclude that for the current acquisition data, *λ* = 13 is the best choice. The ADA at selected ROIs is larger than the global ADA because in those regions, there are obvious edges that contributed to the relatively large ADA values. Compared with the noisy 4-average data, the denoised data show significant improvements. Using the regularization parameter *λ* = 13, the ADA is close to the ADA of the 18-average data. The ADAs of all the ROIs are however reduced compared to the noisy data.

## 4. CONCLUSION

In this work, we have generalized the color TV regularization method of Blomgren and Chan [25] to yield a structure preserving regularization method for matrix-valued images. We have shown that the proposed method performs well as a regularization method with the important property of preserving both edges in the data and positive definiteness of the diffusion tensor. Numerical experiments on synthetically produced data and real data from DTI of a human brain of a healthy volunteer indicate good performance of the proposed method.

## Figures and Tables

**Table 1:** The distance *m*(*D*, D*) of the regularized and the nonregularized tensor fields from the numerical examples shown in Figures 7 and 8.

Averages	*λ*	Reg. *m*(*D*, D*)	Nonreg. *m*(*D*, D*)

2	9	136.1	208.3

4	13	113.5	154

6	19	84.8	105.6

**Table 2:** The average deviation angle (ADA) of the noisy data, the processed data (two different regularization parameters), and the reference data.

Data(↓) ADA (→)	Global	ROI 1	ROI 2	ROI 3

Noisy (4 avgs.)	12.32	32.92	41.02	42.87

Denoised, *λ* = 13	6.27	11.77	31.50	25.27

Denoised *λ* = 20	7.58	13.34	32.88	28.86

Clean image (18 avgs.)	6.65	18.23	24.80	24.80

## APPENDIX

### A. EULER-LAGRANGE EQUATION AND NUMERICAL IMPLEMENTATION

In this appendix, we explicitly write out the Euler-Lagrange equations corresponding to the minimization functional (13). In addition, the numerical scheme used in the simulations in Section 3 is briefly discussed.

Using the short-hand notation

(A.1) $$p\left(x_{ij}\right)=\alpha_{ij}\nabla \cdot \left(\frac{\nabla x_{ij}}{\left|\nabla x_{ij}\right|}\right),$$

we can write out the derivatives of *R* and *F* as

(A.2) $$\begin{array}{c} \frac{\partial R}{\partial \ell_{11}}=2\left(\ell_{11}p\left(\ell_{11}^{2}\right)+\ell_{21}p\left(\ell_{11}\ell_{21}\right)+\ell_{31}p\left(\ell_{11}\ell_{31}\right)\right),\\ \frac{\partial R}{\partial \ell_{21}}=2\left(\ell_{11}p\left(\ell_{11}\ell_{21}\right)+\ell_{21}p\left(\ell_{21}^{2}+\ell_{22}^{2}\right)+\ell_{31}p\left(\ell_{21}\ell_{31}+\ell_{22}\ell_{32}\right)\right),\\ \frac{\partial R}{\partial \ell_{22}}=2\left(\ell_{22}p\left(\ell_{21}^{2}+\ell_{22}^{2}\right)+\ell_{32}p\left(\ell_{21}\ell_{31}+\ell_{22}\ell_{32}\right)\right),\\ \frac{\partial R}{\partial \ell_{31}}=2\left(\ell_{11}p\left(\ell_{11}\ell_{31}\right)+\ell_{21}p\left(\ell_{21}\ell_{31}+\ell_{22}\ell_{32}\right)+\ell_{31}p\left(\ell_{31}^{2}+\ell_{32}^{2}+\ell_{33}^{2}\right)\right),\\ \frac{\partial R}{\partial \ell_{32}}=2\left(\ell_{22}p\left(\ell_{21}\ell_{31}+\ell_{22}\ell_{32}\right)+\ell_{32}p\left(\ell_{31}^{2}+\ell_{32}^{2}+\ell_{33}^{2}\right)\right),\\ \frac{\partial R}{\partial \ell_{33}}=\left(2\ell_{33}p\left(\ell_{31}^{2}+\ell_{32}^{2}+\ell_{33}^{2}\right)\right),\\ \frac{\partial F}{\partial \ell_{11}}=4\left[\left(d_{11}-{\hat{d}}_{11}\right)\ell_{11}+\left(d_{21}-{\hat{d}}_{21}\right)\ell_{21}+\left(d_{31}-{\hat{d}}_{31}\right)\ell_{31}\right],\\ \frac{\partial F}{\partial \ell_{21}}=4\left[\left(d_{21}-{\hat{d}}_{21}\right)\ell_{11}+\left(d_{22}-{\hat{d}}_{22}\right)\ell_{21}+\left(d_{32}-{\hat{d}}_{32}\right)\ell_{31}\right],\\ \frac{\partial F}{\partial \ell_{22}}=4\left[\left(d_{22}-{\hat{d}}_{22}\right)\ell_{22}+\left(d_{32}-{\hat{d}}_{32}\right)\ell_{32}\right],\\ \frac{\partial F}{\partial \ell_{32}}=4\left[\left(d_{32}-{\hat{d}}_{32}\right)\ell_{22}+\left(d_{33}-{\hat{d}}_{33}\right)\ell_{32}\right],\\ \frac{\partial F}{\partial \ell_{31}}=4\left[\left(d_{31}-{\hat{d}}_{31}\right)\ell_{11}+\left(d_{32}-{\hat{d}}_{32}\right)\ell_{21}+\left(d_{33}-{\hat{d}}_{33}\right)\ell_{31}\right],\\ \frac{\partial F}{\partial \ell_{33}}=4\left[\left(d_{33}-{\hat{d}}_{33}\right)\ell_{33}\right]. \end{array}$$

By combining each of the equations

(A.3) $$\frac{\partial G}{\partial \ell_{ij}}=\frac{\partial R}{\partial \ell_{ij}}=\frac{\partial F}{\partial \ell_{ij}},\;\;\;\;\left\{ij\right\}\in \left\{11,21,22,31,32,33\right\},$$

we arrive at the Euler-Lagrange equations corresponding to the minimization problem (13). In the numerical simulations, we use the steepest descent method with a fixed time step. Thus, we have the six equations,

(A.4) $$d_{ij}^{n+1}=d_{ij}^{n}-\text{Δ}t\frac{\partial G^{n}}{\partial \ell_{ij}},\;\;\;\;\left\{kl\right\}\in \left\{11,21,22,31,32,33\right\},$$

where *n* is the iteration index, and Δ*t* is the time-step parameter. We approximate the gradient ∂*G*/∂ *ℓ*_*ij*_ by standard finite difference schemes, see, for example, [4]. We here note that each iteration of the form (A.4) is performed sequentially. Thus, the equations are solved as a coupled system of six PDEs.
